# Retention of medical officers in the district health services of the Western Cape, South Africa: An exploratory descriptive qualitative study

**DOI:** 10.4102/safp.v64i1.5467

**Published:** 2022-05-10

**Authors:** Robert J. Mash, Werner Viljoen, Steve Swartz, Mumtaz Abbas, Leigh Wagner, Herma Steyn, Gavin Hendricks, Dusica Stapar, Andrew Williams, Adeloye Adeniji, Johan Schoevers, Paul Kapp

**Affiliations:** 1 Division of Family Medicine and Primary Care, Stellenbosch University, Cape Town, South Africa; 2 Metro Health Services, Western Cape Government, Cape Town, South Africa; 3 Rural Health Services, Western Cape Government, Cape Town, South Africa

**Keywords:** district health services, district hospitals, primary healthcare, primary care, retention, workforce, medical officers, family physicians, staff satisfaction

## Abstract

**Background:**

An adequate health workforce is an essential building block of effective health systems. In South Africa, medical officers (MOs) are a key component of service delivery in district health services. The Stellenbosch University Family Physician Research Network in the Western Cape identified that retention of MOs was a key issue. The aim of this study was to explore the factors that influence the retention of MOs in public sector district health services in the Western Cape, South Africa.

**Methods:**

This is a descriptive exploratory qualitative study. Medical officers were purposefully selected in terms of districts, facility types, gender, seniority and perceived likelihood of leaving in the next four years. Semi-structured interviews were performed by family physicians, and the qualitative data were analysed using the framework method.

**Results:**

Fourteen MOs were interviewed, and four major themes were identified: career intentions; experience of clinical work; experience of the organisation; and personal, family and community issues. Key issues that influenced retention were: ensure that the foundational elements are in place (e.g. adequate salary and good infrastructure), nurture cohesive team dynamics and relationships, have a family physician, continue the shift towards more collaborative and appreciative management styles, create stronger career pathways and opportunities for professional development in the district health services, be open to flexible working hours and overtime, and ensure workload is manageable.

**Conclusion:**

A number of important factors influencing retention were identified. Leaders and managers of the healthcare services could intervene across these multiple factors to enhance the conditions needed to retain MOs.

## Introduction

The Sustainable Development goal 3, part c, emphasises the need to ‘substantially increase health financing and recruitment, development, training and retention of the health workforce in developing countries’.^1^ The healthcare workforce is one of the essential building blocks of effective health systems.^2^ Health systems must ensure that they have a sufficient number of healthcare workers with a combination of junior and senior staff to deliver services. Effective health services depend on the availability, competence and motivation of healthcare workers.^3^ District health services, including primary health care, are the foundation of effective health systems.^4,5^ In low- and middle-income countries (LMICs), district health services rely on community health workers, nurses, physician assistants and doctors.

Low- and middle-income countries, particularly in Africa, have a scarcity of human resources for health.^6^ Doctors are often maldistributed in urban areas and serve more affluent communities in private practice.^7^ In South Africa, doctors without a specialisation in the public sector are referred to as medical officers (MOs), while in the private sector they are referred to as general practitioners. The public sector looks after about 80% of the population.^7^ District hospitals rely on MOs, while primary care is mostly delivered by nurses, with support from MOs. Therefore, MOs are the backbone of service delivery in the district hospital and essential members of the primary healthcare team. District hospitals are often rural and remote, and the recruitment and retention of MOs in these locations are particularly challenging and important.^8^ Although interns and community service MOs are obligated to spend time in the district health services, there are concerns as to whether the services can retain MOs on a more permanent basis.^8,9^

Attracting and retaining MOs in district health services is a complex problem. Most research has focused on health professionals in rural and remote areas,^10,11^ and factors identified for rural retention include adequate income and incentives, appropriate workload, access to specialist care and outreach, availability of continuing education, spouse career opportunities, acceptable education for children, acceptable living and working conditions, safety, opportunities for career advancement, and recognition by managers, peers and patients.^12^ Financial rewards, career development, continuing professional development, adequate infrastructure and resources, effective management and recognition have been identified as important factors in retaining the health workforce in LMICs.^13^ In Ghana, motivation and job satisfaction were related to retention or turnover of staff in district health services, and the corollary of this is that job stress and burnout are important.^14^ An additional issue related to retention amongst British general practitioners is the need for part-time and flexible working hours, particularly with the feminisation of health care and needs of mothers with young children.^15^

A recent ministerial task team on human resources for health in South Africa was unable to make any recommendations about MOs at district hospitals, but suggested there would be a gap of 2293 non-specialist medical practitioners in primary health care by 2025.^8^ The same strategy document emphasised the importance of retaining the health workforce, particularly in rural areas. In 2019, there were 33.1 medical practitioners per 100 000 population (range between provinces of 25.9–45.3 per 100 000) and 10.0 specialists per 100 000 (range 1.4–25.8 per 100 000). By 2025, the goal was to improve equity between provinces and have 37.8 medical practitioners per 100 000 and 15.2 specialists per 100 000.^8^ The proposed legislation for National Health Insurance also emphasises the need to address retention of the workforce.^16^ They suggest a number of factors that may be important, such as job design, performance management systems, remuneration policies, employment relationships, infrastructure, workplace culture, resources, workforce planning and career pathways.^16^

In South Africa, it has been possible to specialise as a family physician since 2007 and remain in the district health services as a specialist, thus offering a new career path for MOs. However, human resources for health policy has not fully understood or supported the contribution of family physicians and opportunities are highly variable between provinces.^17^ Specialising in family medicine is now a viable option for MOs who want career progression, while remaining as a generalist in the district health services. The Western Cape has embraced the deployment of family physicians in district hospitals and community health centres.^18^

In the Western Cape, a research network of family physicians, linked to Stellenbosch University, prioritised the need to evaluate factors influencing the retention of MOs in district health services. The 2020–2021 Western Cape Government’s annual report on health found a vacancy rate of 5.9% for posts in the district health services, although no specific figure was given for MOs.^19^ They also commented that the vacancy rate was linked to their ability to fund all the available posts. This vacancy rate, however, does not determine what the turnover of staff is and how well individual MOs are retained in the system. Continuity of service from an individual MO over many years is likely to add more value than a MO post that is continuously re-filled. Family physicians, who are the lead clinicians at their facilities and responsible for clinical governance, identified this as a knowledge gap in the local context. Addressing this gap could help identify interventions to improve retention. Although many of the factors influencing retention are known, their relative importance in different contexts needs to be determined.^13^ The Stellenbosch University Family Physician Network (SUFPREN) identified a number of factors significantly associated with retention of MOs in a previous unpublished survey. These factors were older age and higher MO grades, long-term career plans, the overall rating of the facility, feeling supported in the workplace, staying with one’s partner and having good educational opportunities for children. The aim of this study was to explore these and other factors that may influence the retention of MOs in public sector district health services in the Western Cape, South Africa.

## Methods

### Study design

This was a descriptive exploratory, thematically analysed, qualitative study, which explored factors influencing the retention of MOs in the district health services of the Western Cape.

### Setting

**FIGURE 1:**
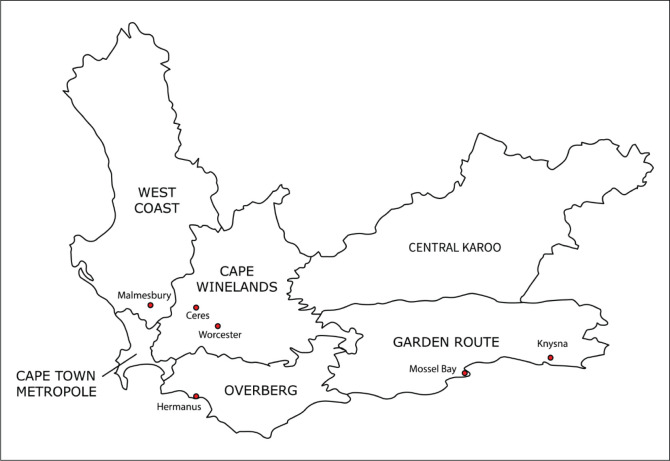
Map of the district and sub-districts of Western Cape province.

District health services in the Western Cape are offered via one metropolitan district that covers the City of Cape Town and five rural health districts (Figure 1). District health services are offered via district hospitals, community health centres and clinics. Medical officers are employed at district hospitals and community health centres.

District hospitals typically have adult male, adult female, child, maternity and labour wards, as well as an operating theatre and outpatients. In addition, district hospitals will have an emergency centre that receives referrals and walk-in patients from the primary health care platform. Medical officers typically work in one of these wards for a period, and then rotate between different wards. All MOs will work in the emergency centre and cover the whole hospital when doing overtime. During this study, many services were reorganised because of the coronavirus pandemic. So, for example, the adult male and adult female ward may have changed into a ward for those suspected or known to have coronavirus and a ward for those not suspected to have coronavirus. There is a hierarchy of MOs from interns, community service MOs, grades 1–3 MOs and finally the specialist family physician as the most senior clinician. Medical officers progress from grade 1 to grade 3 with every five years of service. In some facilities, there are also registrars in training to become a family physician. More senior MOs tend to develop areas of interest and may spend more time in one part of the hospital, for example, the operating theatre, labour ward, adult or child medicine. Medical officers are often also responsible for outreach and support to the primary health care platform, and some also develop a special interest in this aspect of the work.

Community health centres typically have a team of primary care providers, and most consultations are with a nurse practitioner. Health centres see people across the life course, burden of disease, and offer promotive, preventive and therapeutic services. Many health centres also offer palliative and rehabilitative care. Some health centres are open 24-h a day and may have an emergency centre and a midwife-led obstetric unit. Medical officers are responsible for supporting the nurse practitioners and seeing more complicated patients. For example, MOs may see people with poorly controlled chronic conditions or multimorbidity. The family physician at these health centres is again the most senior clinician and usually sees patients referred to them by the MOs and nurses. The MOs at community health centres may also provide outreach to the smaller clinics in the sub-district. The facility-based services also support the community-based teams of community health workers.

### Sample size and selection

The SUFPREN includes family physicians from the district health services that are linked to Stellenbosch University.^20^ This study was an initiative of SUFPREN. This network enables them to participate in collaborative applied research projects. Family physicians identify the key research questions and collect data within the network whilst researchers at the university assist with methodology, analysis and report writing. Family physician from 14 facilities throughout the Western Cape were involved in the study (Table 1). Facilities were equally split between rural and metropolitan areas and covered five districts in the Western Cape.

**TABLE 1:** Characteristics and location of the study sites.

Name	District	Number of beds (DH)/mean monthly headcount (CHC)	Number of interns	Number of COSMOs	Number of registrars	Number of medical officers	Number of family physicians
Kraaifontein CHC	Metro	20 000	8	1	0	7	1
Elsies River CHC	Metro	11 000	4	1	0	6	1
Khayelitsha CHC	Metro	24 000	3	2	0	10	1
Bishop Lavis CHC	Metro	9000	3	0	0	4	1
Michael Mapongwana CHC	Metro	18 000	2	2	0	5	1
Eerste River DH	Metro	150	12	4	4	13	3
Helderberg DH	Metro	181	24	6	8	13	4
Ceres DH	Cape Winelands	106	0	5	2	6	2
Stellenbosch DH	Cape Winelands	85	3	2	2	6	2
Worcester CHC	Cape Winelands	7525	1	3	0	5.5	0.5
Mossel Bay DH	Garden Route	90	4	2	2	9	1
Knysna DH	Garden Route	90	4	6	2	5	2
Swartland DH	West Coast	86	0	3	3	6	2
Hermanus DH	Overberg	71	3	3	1	7	1

DH, district hospital; CHC, community health centre; COSMO, community service medical officer.

**FIGURE 2:**
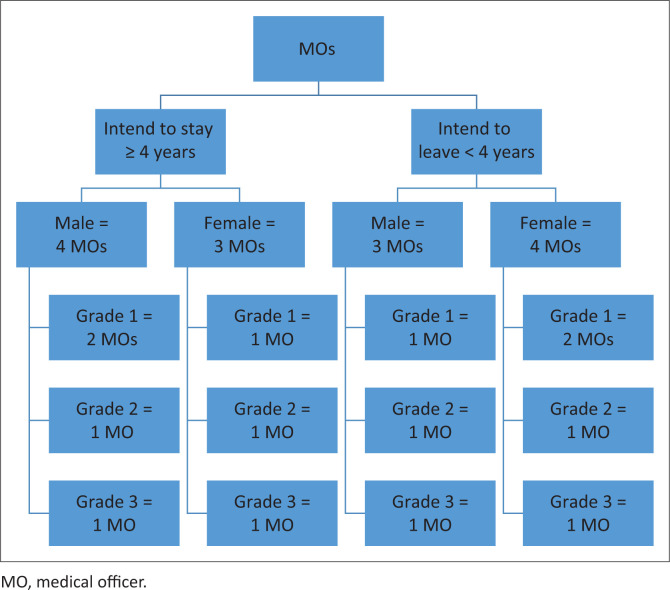
Sampling strategy for medical officers.

One of the MOs was purposively selected from each facility to give maximum variation in the sample. Medical officers were selected based on gender, grade and whether the family physician thought they were likely to leave within the next four years or stay (Figure 2). The family physicians lead these clinical teams, work closely with all the MOs, undertake their performance appraisals, and have a good idea of which MOs are thinking of staying or leaving the facility. Community service MOs and interns, who did not have a permanent contract at the facility were excluded. The family physicians suggested that a 4-year threshold was a practical divide between those who might stay long term and those who were likely to move on in the near future. This was seen as a more rigorous threshold than just looking at who would be retained in the next year.

Family physicians were asked to identify two MOs at their facilities, according to the criteria in Figure 2, who could be interviewed. The lead researcher (R.M.) then made the final selection of MOs to ensure that all facilities and all sections of the grid were included. A family physician from a different facility, who was responsible for interviewing that MO, then made contact to obtain consent and set up the interview. It was thought that this sample would achieve saturation of themes; however, further interviews could be undertaken if the analysis suggested that themes needed further exploration.

### Data collection

A semi-structured interview guide was developed based on issues from the literature and the preceding survey of MOs.^21^ The opening question was as follows:

How likely is it that you will continue working here (i.e. in this facility) over the next few years and what are the main factors influencing your decision to stay or go?

The guide then suggested questions to explore the following areas:

- Professional issues: co-workers, clinical support, mentoring, job content, having a family physician, management, recognition and meaning and career pathway.
- Financial issues: contract, remuneration and overtime
- Personal and family issues: partner, family and personal issues

Each family physician was linked with one MO at a different facility. They conducted and recorded interviews in English via the internet using MS Teams or Zoom. Interviews lasted between 30 min and 60 min.

Retaining the MOs at their facilities was important to family physicians as this directly impacted the quality of care and stability of service delivery. While they were aware of issues affecting retention from their own experience, they all followed the same interview guide to explore the same topics with the respondents. Family physicians interviewed MOs from a different facility, where they did not work. During their postgraduate training, family physicians were trained in communication and interviewing skills. The interview process and guide were workshopped with the family physicians. The family physician interviewers were seven men and seven women, with all having a Masters of Medicine degree.

### Data analysis

The recordings were transcribed verbatim, checked for errors and uploaded in Atlas-ti software (version 8) for analysis by the first author (R.M.). Thematic analysis used the framework method:^22^

- Familiarisation: The researcher listened to the first seven tapes and listed key issues or ideas that emerged from the data in order to become familiar with the data and to inform the development of the coding index.
- Construction of coding index: The researcher defined a list of codes that were derived from the issues and ideas in step 1. Similar codes were organised into categories in the coding index.
- Coding: The researcher used the coding index to systematically code the data across all transcripts in Atlas-ti. Additional codes were added if new issues emerged that were not in the index.
- Charting: The researcher generated reports in Atlas-ti that brought all the data on a single code together for interpretation. The report included the code definition, any comments made during analysis and the origin of each quote included in the report.
- Mapping and interpretation: The researcher read the reports from step 4 and interpreted the data, looking for the range of experiences and opinions within each theme and any relationship between themes. The themes were interpreted inductively from the data contained in the reports.

The themes emerging from the data were found across multiple interviews and the last few interviews that were coded did not identify new themes. The analysis was presented to all the family physicians that conducted interviews at a workshop for peer review and validation of the interpretation. The family physicians at the workshop and the researcher who conducted the analysis did not identify any themes that required further interviews. The research team were, therefore, satisfied that reasonable saturation of themes was reached.

## Findings

Fourteen MOs were interviewed, and their characteristics are shown in Table 2. Overall, eight were in the rural health services and six in the metro health services; nine were based at district hospitals and five were in health centres. Nine were female and five were male; seven were grade 1, four were grade 2 and three were grade 3 MOs. Seven were planning to leave in the next four years and seven were planning to stay longer. It was not possible to interview an MO at Michael Mapongwana CHC, and therefore, two MOs were interviewed from Stellenbosch DH.

**TABLE 2:** Characteristics of medical officers.

Source	Location	Facility type	Sex	Age (years)	Home language	Job grade	Years working at facility	Intention
1	Rural	District hospital	M	35	English	1	2	Leave
2	Rural	District hospital	F	40	Afrikaans	3	15	Stay
3	Rural	District hospital	F	34	Afrikaans	2	6	Stay
4	Rural	District hospital	M	32	Afrikaans	2	3	Leave
5	Metro	District hospital	F	32	English	2	7	Leave
6	Rural	District hospital	F	40	Afrikaans	3	2	Stay
7	Metro	Health centre	F	28	English	1	3	Leave
8	Metro	District hospital	M	30	Afrikaans	1	2	Leave
9	Metro	Health centre	M	50	French	3	1	Leave
10	Rural	District hospital	M	37	Afrikaans	2	6	Stay
11	Metro	Health centre	F	31	English	1	5	Stay
12	Metro	Health centre	F	28	English	1	1	Leave
13	Rural	District hospital	F	31	Afrikaans	1	2	Stay
14	Rural	Health centre	F	31	English	1	5	Stay

F, female; M, male.

The findings are presented as four main themes:

- Personal career intentions
- Experience of clinical work
- Experience of the organisation
- Personal, family and community issues.

In the analysis, there did not seem to be a clear differentiation of themes between those who intended to stay versus leave. These groups are therefore not juxtaposed in the findings.

### Personal career intentions

#### Ambivalence about specialising

Working in the district health services was considered as a good way to improve one’s skills while keeping many options open for future specialisation. Many of the grade-1 MOs expressed uncertainty about their future career and were still trying to identify their passion. A wide variety of options were being considered from private general practice, specialisation, management, teaching and research. One MO was also considering leaving medicine entirely:

‘So it’s a constant battle in my mind because I don’t really know where I want to end up. I know that I can’t do this forever.’ (Number 13, district hospital, grade 1)

Medical officers who preferred a wide variety of clinical work and exposure to multiple disciplines were more likely to stay in the district health services, while those who were attracted to one specific discipline or who wanted to avoid exposure to certain clinical areas were more likely to consider leaving or specialisation. Medical officers commented that people had left the one hospital that did not provide a full scope of practice. Larger district hospitals allowed MOs to focus more on one clinical area, which might encourage those more interested in one clinical area to remain. However, MOs at these larger hospitals might also be challenged to manage patients more appropriately to higher levels of care and outside their comfort zone:

‘So the biggest reason [*to stay*] is actually just the type of work that we do, I enjoy the district level primary care setting, being able to be in the hospital, and seeing a wide variety of different patients and problems, but also from different departments.’ (Number 4, district hospital, grade 2)

The desire to leave and specialise in some MOs was in tension with the desire to stay working in the district health services, enjoy a rural lifestyle or avoid the disruption of parenting that being a registrar might cause:

‘Yes eventually, probably, like I want to do a surgical field -orthopaedics, reconstructive, like to probably specialize, but also to specialize I have to move to a city, I have to move out of the environment. So I am quite happy where I am, I think this is a good place to raise children.’ (Number 10, district hospital, grade 2)

Although MOs progressively became more competent and were promoted with years of service, the nature of the work remained the same. Leaving the district health services to specialise was seen as a way of improving one’s status, being more autonomous and mastering a particular field of medicine:

‘I felt that in the district health system I could do a lot of courses that would improve my skill but in terms of improving my level of workmanship I felt that I wasn’t growing and that I was essentially … you know yes, I was growing in terms of a pay grade, but that wasn’t what I wanted you know for myself. I wanted to grow in terms of the level of experience that I have.’ (Number 5, district hospital, grade 2)

Grade 3 MOs were not usually considering a major change of career or specialisation. There was a sense that a combination of inertia, family commitments and being comfortable in the district health services had reached a point at which radical change was unlikely:

‘I think I am in a comfort zone now. That’s probably bad but I’ve never thought of like specialising. I think when I finished I got married. And two years later, I got kids. And you know, stuff like that in your work, it’s difficult, so I take my hat off to you guys that with small kids and stuff like that still goes and studies and stuff, that has that ability to do that still because I don’t know how you guys survive.’ (Number 2, district hospital, grade3)

#### Not sure about family medicine

Few of the MOs were actively considering a career in family medicine. Some were clearly more interested in developing advanced skills in a specific area through specialisation, and a few felt that they were too old. Several were put off by the need to train in disciplines that they were not comfortable with such as surgery, obstetrics, anaesthetics, paediatrics or psychiatry. Several were concerned that the lack of family physician posts meant they might not be employed on qualifying. In addition, they might give up a permanent MO post for a temporary registrar post, with no guarantee of being able to stay at the end of their training. In rural areas, some of the MOs who were interested could not get a registrar post. One or two felt that family physicians spent too much time on administration and management and not enough time with patients:

‘The other reason is probably if we just look at kind of the trend of what’s happening in the health, The Health Department with regard to there’s not a lot of family medicine and consultant posts available. You see a lot of the registrar’s that finish up ending just in the MO post again.’ (Number 8, district hospital, grade1)

‘I was scared for the entire family medicine dynamic, it’s very diverse. You have to be a very good all-rounder and I’m not … I’m owning up to it … I’m not good at surgery, I’m not good at psychiatry and I think the one thing that I’m really good at is internal medicine. I love studying it, I love being a part of it, it’s what I would live, breathe and do every day.’ (Number 5, district hospital, grade 2)

‘And I wasn’t willing to give up my MO post and not being assured of a family medicine post once I’m done, because I really wouldn’t like to move.’ (Number 6, district hospital, grade 3)

#### Not keen on private sector

Only a few MOs were seriously considering a career in the private sector, and most preferred to stay in the public sector. They felt that they made more of a difference to the society and saw a wider variety of problems, which allowed them to develop more. In the public sector, there was also the opportunity to care for a patient in both primary care and district hospital. While you had to work hard in the public sector, MOs appreciated the predictable salary and benefits. They felt that you might have to work harder in the private sector to earn the same salary and then also had to deal with running a business. However, one could control one’s own working hours more. One of the MOs felt uncomfortable with the unequal quality of care offered in the private sector to those with and without insurance. There was also a view that the management plan was more influenced by patients’ demands and the business imperative, rather than evidence. However, one or two MOs thought that the private sector was attractive because the overall environment was better resourced, safer and more controlled:

‘I think it’s quite fair. I have compared it with private practice as well. And if you take into account all the overheads and extras that you incur in a private practice, I actually think we, we are getting fair remuneration in terms of private practice, with, for me the added bonus of not having to worry about medical aids and staffing and locums and those types of stuff.’ (Number 6, district hospital, grade 3)

‘In the private setting of course you will have that much more control over not just the number of people that you see but also the boundary will be much more firmly in place you know, you’ll be, you’ll be able to see patient without being interrupted or without being sort of shouted at down the corridor or something.’ (Number 12, health centre, grade 1).

### Experience of clinical work

#### Being part of a great team

Maybe, the strongest sub-theme in deciding to stay was the quality of teamwork and relationships with colleagues. The overall impression was that a cohesive and supportive team was a critical factor in retention of MOs and might compensate for many other negative factors, while an unsupportive and unhappy team might have the opposite effect. The perception was that interns and community service MOs wanted to be re-employed at facilities that had good teamwork. Medical officers spoke about working within their ‘comfort zone’ when they were part of a well-functioning team:

‘And the biggest factors are just that I’m comfortable where I am. I have a new community if I can put it that way … I know the people who are working here, and I have good camaraderie with all my colleagues going from the doctors down to the cleaners. And … that’s mostly the reason why I’m still here.’ (Number 3, district hospital, grade 2)

In general, MOs were very positive about the teams that they worked in and felt that people worked hard, shared the load and supported each other. Medical officers contrasted their positive experience of teamwork in the district health services of the Western Cape with more negative experiences elsewhere during community service and at specialised or tertiary hospitals. At district hospitals, people commented on how the team included everyone from the porters and cleaners to nurses and doctors. In some rural areas, their colleagues became friends and even felt like family:

‘I think, the main factors that keep you in a small district level hospital, like mostly teamwork, I think if you work in a, quite a good team, in a smaller hospital, most people are friendly, they know each other, it’s more intimate, it’s actually a lot better than working in a big hospital with lots of consultants, with different departments where you get departmentalised.’ (Number 10, district hospital, grade 2)

Key aspects of this teamwork included getting to know everyone by name, accommodating each other’s needs, having complimentary skills, being respectful and collegial, having open communication, solving problems together, and being clear about different roles in the team and fulfilling those roles. Teams were not always perfect and MOs spoke about the pressures encountered during the COVID-19 pandemic, individuals bringing negative emotions to work, miscommunication and loss of cohesion from turnover of staff:

‘But I feel the team sometimes is a bit disjointed. I think with COVID-19 obviously, people are taking strain. So it’s not always like intentional, but sometimes there’s a bit of miscommunication. And people, you know, negatively affect each other, because of probably what’s going on at home or, you know, so … I get affected by how other people are feeling and that type of thing.’ (Number 13, district hospital, grade 1)

#### Good support and supervision

Overall, MOs felt that they could always obtain help with specific procedures. They looked to grade 3 MOs, senior registrars and family physicians to provide this clinical support. Senior clinicians were approachable and gave constructive feedback to help MOs improve their clinical management. In some district hospitals, there was a clear division of skills, for example, one MO would be the expert on orthopaedics, while another would know obstetrics:

‘Well, usually when I have a complaint, or if I have any questions regarding patients, if I’m not too sure, I usually go to her office [*the family physician*]. If she’s busy in her office, or I don’t go to her office, I usually give her a call and she always responds or answers.’ (Number 12, health centre, grade 1)

A lack of support for junior MOs was seen as a key reason to leave, because it was stressful to feel out of one’s depth and make unnecessary mistakes. Such experiences could erode confidence and motivation in the longer term. It appeared that support had improved tremendously over the last few years, although MOs did not like relying on locums or local GPs for support. Having sufficient senior clinicians in the team created a supportive, safe and yet challenging environment in which people could learn:

‘When we get new doctors starting or Comserves and stuff like that, … the family physicians ask them on “what are their shortcomings” and “what they need to do to improve”. So the environment has really changed, where I got from till now. And that’s why as I said in the beginning I didn’t want to stay, I wanted to leave to be honest, because of the attitude here …’ (Number 2, district hospital, grade 3)

#### Good outreach and linkage to the next level of expertise

Medical officers were also appreciative of support from specialists at the local referral hospital. Most facilities had some kind of outreach where specialists visited the facility to see patients and at the same time would conduct ward rounds, provide clinical training or continuing professional development. Although such visits stopped during the coronavirus pandemic, there was still telephonic support, virtual training (via zoom) and interaction on emergency patents via the Vula app:

‘In surgery the same thing, we do have visiting consultants coming for rounds, and coming for like theatre days. So, you know, there was always a matter of being able to, if you really, if you’re really stuck on something, you know in the next day or so there’ll be a consultant to review the patient with you.’ (Number 8, district hospital, grade 1)

Despite the benefits of supportive outreach, several MOs spoke about the frustrations of trying to refer patients and being inappropriately expected to manage patients at the district level:

‘And a very clear example is intubated patients coming to our emergency centre and then [*the*] hospital refuses to take the patient, and then you sit with an issue of you’ve got an intubated patient within our casualty, and we were not equipped to manage that and then that is a big problem.’ (Number 2, district hospital, grade 3)

#### Having a family physician

The family physicians were regarded very positively and fulfilled a number of different roles. They supported the MOs clinically as consultants and created a safe working environment. Sometimes, senior registrars or family physicians working in MO posts felt more approachable or available to the MOs. Having family physicians meant that the team could handle more complicated patients and avoid referrals to the next level of care. They were also mentioned as clinical role models who encouraged a more wholistic approach:

‘It makes a huge difference … I did work in a facility where there was no family physician … and you can see the way that clinic were running. It was a poor clinic I can say, if you can compare with this clinic where there are two family physicians, you can see the input of those two family physicians in the management of this clinic. This is very, very good … [*A*] very good thing to have a family physician in the clinic.’ (Number 9, health centre, grade 3)

Family physicians were also considered as role models for clinical training and created a learning environment, in which MOs likewise supported more junior colleagues. Family physicians provided clinical training and teaching and encouraged MOs in their professional development. Usually it was the family physician who conducted performance appraisal during which such development was discussed. Family physicians also attracted registrars and medical students into the facility. They were frequently mentioned as being appreciative when providing feedback to MOs:

‘The family physician will tell you there is an appendix, let’s go and do it, you are going to do it. And then it’s actually, step by step, teaching you explaining the procedure beforehand, asking you what you want to do, how are you going to approach it and then at the end, it’s like, okay, this was nice.’ (Number 10, district hospital, grade 2)

Family physicians were also observed for their ability to improve service delivery and processes within the facility and to advocate for important clinical issues. A few family physicians were criticised for being too distant and absorbed by administrative tasks. At district hospitals, some of the family physicians who were more dedicated to primary care were no longer seen as competent in the hospital skills set. Although having a family physician was not seen as the most critical factor in retaining MOs, it contributed to the intention to stay:

‘The family physicians at this hospital are making the decision to leave very difficult because it’s very bittersweet. They encourage me so much to pursue what I feel I’m good at or what I feel I allow. But because they’re so brilliant and so well put and so cohesive it makes it very difficult to leave them.’ (Number 5, district hospital, grade 2)

‘To be honest our family physician specifically, she’s not that much on the ground if I can put it that way. She’s more in an administrative role in my opinion. So I don’t think she’s specifically the determining factor of whether I would stay or not.’ (Number 3, district hospital, grade 2)

#### Recognition by patients and peers for clinical work

Appreciation and recognition of their work by patients were mixed, and there was an impression that this might be commoner in rural areas. Some of the junior MOs were quite frustrated at the way patients interrupted consultations and could be rude or disrespectful. This frustration could contribute to a desire to move to a different type of work environment:

‘I think it’s things like you will be busy with the patient and they will stand at the door and look at you and call you, or they will be like hey, girl! Or Meisie! So it’s like are you talking to me? So it is little things like that.’ (Number 12, health centre, grade 1)

Most MOs found their work meaningful and felt that they were making an important difference, although their contribution was not always appreciated by peers working in referral hospitals. A perception that one’s work was not respected by one’s peers could contribute to a desire to move on:

‘A few weekends ago I met up with friends and you know all our varsity friends and everybody is basically on their own different paths. And I did feel that there was a sense of like oh my word like you’re just an MO at the CHC like what difference are you making? … So I do feel that this work is very meaningful, but I do think … it’s not very rewarding.’ (Number 7, health centre, grade 1)

### Experience of the organisation

#### Good salary and benefits

Medical officers were happy with their remuneration and felt that this provided them financial security. They noted that the salary increased progressively and was the same throughout the public sector, so moving somewhere else would not change the package. The rural allowance was observed as an added benefit for some MOs. They differed in their opinion as to whether colleagues in the private sector actually took home more at the end of the day:

‘I feel like the amount I’m getting paid, I’m happy with it, I’m not feeling like I’m working too, how can I put it, I don’t feel like the ratio is off, that I am getting paid too little for what I am doing. I’m happy.’ (Number 8, district hospital, grade 1)

#### Overtime is essential, but needs some flexibility

Medical officers regarded overtime as stressful, but as a necessary part of the job and an important source of income. Medical officers were more likely to leave facilities that were not willing to be flexible with overtime commitments, particularly in relation to parenting and children. Medical officers also appreciated facilities that allowed them to change the nature of overtime as they became more senior, for example, being on call from home to cover junior colleagues:

‘Overtime, it is very time and energy consuming. At some point, you’re going to start a family. That is for me more important than, than earning more money or working, you know working very hard. And so if there’s not a possibility for me to resign my overtime that will definitely be a reason why I leave yes.’ (Number 4, district hospital, grade 2)

#### Collaborative, responsive and appreciative leadership is needed

Overall, most MOs were quite happy with the management style, and there was the impression that this had improved over the last few years. Most current problems with management were in the community health centres and not the district hospitals. At several district hospitals, family physicians had become part of the management team, particularly as clinical managers. Medical officers reported that their managers were approachable, responsive, supportive, and solved problems and were reasonably efficient. The performance appraisal process provided regular opportunities for MOs to give and receive feedback on any problems:

‘So we’ve got the family medicine physicians here and the one also works as a clinical manager. And we’ve got one CEO [*chief executive officer*]. I have never felt like I could not go to any one of them or even be on WhatsApp terms with any one of them. They are available literally 24/7 and even more so in the pandemic. If I have any questions, any personal concerns, all of them have an open-door policy so the support and from the supervisors is there.’ (Number 5, district hospital, grade 2)

There were a few negative experiences of management, which could influence MOs to leave. Specific issues included rigidity towards change, inability to confront problems, not taking responsibility for a problem, being authoritarian or aggressive and victimising MOs:

‘A lot of the times they, if it’s a certain issue, the management sometimes has already made up their mind of how they are going to deal with it, or what they want to do with it. And then they’ll ask us for suggestions. And then when suggestions are given the idea, the suggestion is really like shut down so aggressively, that it makes people not actually want to participate.’ (Number 12, health centre, grade 1)

Almost all the MOs described an organisational culture in which they were acknowledged and appreciated for their contribution by family physicians and managers, while not a critical factor such acknowledgement did appear to encourage retention. At the same time, MOs felt that their status in the health system was lower than colleagues working at referral hospitals:

‘If you do something well, it is recognized and acknowledged. And to be honest, that’s very important. Like if you constantly work in an environment where you can be graded or criticized, it’s going to be debilitating for your mental and your emotional health, which will have a direct influence on if you want to stay on in a place or not.’ (Number 4, district hospital, grade 2)

#### Good staff numbers and mix are needed

A number of MOs described how the staffing levels and mix had improved along with the workload and working conditions. They described a well-functioning mix of expertise from interns, community service MOs, to permanent MOs (sometimes with special interests) and family physicians. Although community service MOs might want to stay, there were not enough posts to retain them. The team could be disrupted by turnover of staff and too many doctors taking maternity leave, which forced the use of locum doctors. One or two facilities appeared to be short-staffed, which put stress on the team and increased the likelihood of people leaving:

‘I think our team in general has a few seniors probably with 10, 15 years plus experience, like probably three or four of them. And then we’ve got a few grade 2 MOs, on the same level that I’m currently working at. And then we’ve got a lot of junior, so we do have a lot of Comserves. I think six doctors out of 15 or 20 doctors are Comserves. And then four interns, so we’ve got quite a lot of senior people.’ (Number 10, district hospital, grade 2)

‘We’ve recently had a lot of people coming and going, and people are leaving and contract workers. And that has also placed a lot of strain on the team. So I think that yes, and we don’t have enough people and so on. So I think that also affects, you know, how I feel towards the team.’ (Number 13, district hospital, grade 1)

#### Manageable workload is important

Medical officers perceived that the daily workload was on a spectrum from ‘manageable’ to ‘overwhelming’, but no one was planning to leave because of the workload. Key factors that influenced the workload were the cohesiveness of the team (helping each other out), the staffing levels, the way patients’ flow was organised and the variation in activities from day to day. The relentlessness of the workload and the fact that it did not get better as you became more senior was perceived as a negative factor:

‘The workload distribution during the day I think is quite fair as well. Everybody also rotates through the different areas and gets exposed to every area so if you’re in a clinic like I am today, … Clinic, it can get very busy, but tomorrow you are at the … Clinic again and there’s a little bit of reprieve there.’ (Number 6, district hospital, grade 3)

#### A rich learning environment

Medical officers described a rich learning environment in which teaching and training were role modelled by family physicians and cascaded downwards throughout the team with MOs supporting those more junior. As a result, MOs reported that they were constantly confronted with new challenges and learnt in a supportive environment. They were gradually given more responsibility and encouraged to set learning goals for themselves and to study further or attend courses, which were paid for by the facility. One or two MOs commented that the learning environment at the district hospital was better than at the tertiary hospital:

‘And the reason why I think I still stay here is because at the moment, I’m still, you know, every day, I’m still learning a lot, and I’m getting a lot of senior supervision from either the consultant, the visiting consultants that we have, or from the family physicians that we have at the hospital.’ (Number 8, district hospital, grade 1)

#### Adequate supplies, equipment and infrastructure

Medical officers complained about the temporary lack of some equipment and supplies; however, this was not perceived as a major problem. The lack of medication could cause friction with patients; however, the situation in the Western Cape was perceived as much better than elsewhere. There were no complaints about the infrastructure and several MOs commented on recent improvements:

‘It [*stock outs*] really varies from one month to the next what we have and what we don’t have. And, in general, you can make do with what you have. So I don’t think I would leave just because we don’t have something for a short period of time.’ (Number 13, district hospital, grade 1)

### Personal, family and community issues

#### Being with your partner

A very clear reason for leaving was to be with a long-term partner. Partners could influence how MOs felt about specialisation or require them to move to accommodate their own needs. Retaining a MO might also depend on how flexible the organisation was with overtime and contractual obligations to accommodate family life, childcare and the partner’s work commitments:

‘Yes, I think it’s very important. Because I mean, obviously, you’re not, I’m not just by myself, I am with my partner, with my two kids, with my mom, and the rest of my family. But definitely, regarding my immediate family, I think it’s very, very important. I mean, they, they influence us, or they influence me. And I think what they, what they think about my career and my choices that I make is whether it is in public or private, I think it is very, very important. And they play a big role in that decision.’ (Number 11, health centre, grade 1)

#### Prioritising parenting and childcare

Many MOs recognised the importance of parenting and childcare in their lives. Overtime and on-call commitments could at times directly compete with parenting and childcare responsibilities. A lack of flexibility to accommodate MOs’ needs at this point in their lives could lead to them resigning. Several MOs commented that they would stay because the local community had excellent schools and a child friendly environment. One MO felt the private sector would be attractive because you could determine you own hours of work to dovetail with childcare:

‘I actually ended up taking them out [of childcare] because I was working, like having trauma days at those times, and I would end up finishing here like at ten past five but she needed to be fetched by five o’clock already. The teachers ended up getting upset with me because they needed to go home…I will definitely feel influenced in terms of wanting to rather go and just having more time with my family instead of, you know, dedicating so much time to my career.’ (Number 11, health centre, grade 1)

#### Being close to family

Having family close by so that you could regularly visit was an important factor in retaining MOs. Families helped MOs to debrief, helped with child care and were emotionally supportive. Sometimes, MOs needed to also take care of older parents. Family ties were clearly a key factor for many MOs in their choice of location:

‘One of the reasons why I left … Hospital is that there was not a very good social network, environment or support structure as all my family and friends were actually in Cape Town. So when I moved to … that changed my demeanour towards working to a great extent, because suddenly I’ve got loved ones close by. And it also just, you know, when you’re not working, it helps to us to give your perspective or what’s actually happening in the world, kind of what’s important and what’s not.’ (Number 4, district hospital, grade 2)

#### Positive about local community

Medical officers were also influenced by the community and environment where they lived. One of the MOs had grown up in the community and felt like they were giving back. Several MOs expressed appreciation for good amenities, schools, accommodation, social opportunities and an active lifestyle. Safety and security was also an issue. Not having such a connection might prompt a desire or willingness to leave:

‘Good schools, school kids, like I said, best run municipality in the country for, that’s always an added bonus … I say the afterhours, all the social, you’re living close to the beach, you’re living, you can go to the beach whenever, you can lots of outdoor activities, you can join in hiking, running, whatever you feel like doing.’ (Number 6, district hospital, grade 3)

Living far from the hospital and travelling to work was also an issue for several MOs that made it unlikely they would stay in the long term. On the other hand living close by and having easy access to the hospital when on-call and the ability to quickly go home or sort out children was a positive factor.

Across all the themes, the key factors that were spontaneously mentioned as retaining MOs were: good relationships within the clinical team, the amount of support and clinical supervision, the positive learning environment, the variety of meaningful clinical work, the good salary and benefits, a manageable workload, being close to family, living in a good location and not having to travel too far to work.

## Discussion

**FIGURE 3:**
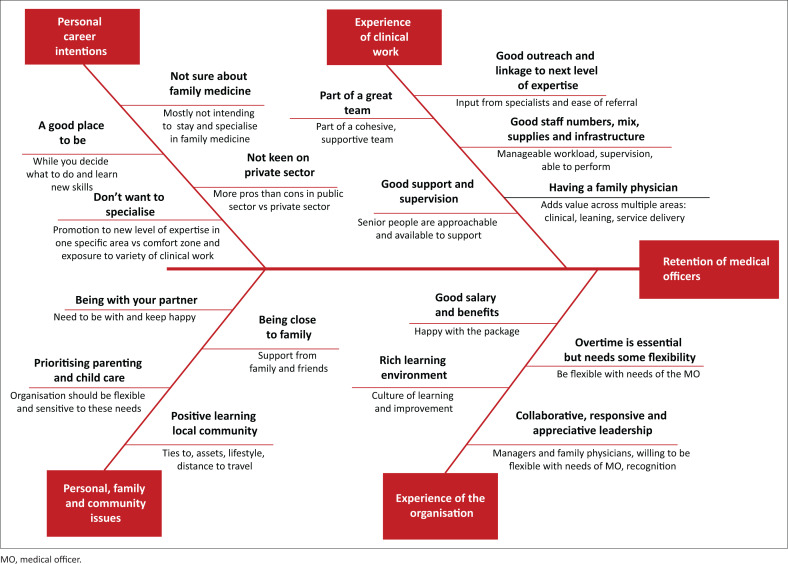
Summary of key findings.

The key findings as a fishbone diagram of themes and sub-themes related to retention of MOs in the district health services are summarised, as shown in Figure 3.

The findings resonate with the results of the preceding survey and shed some light on the factors involved.^21^ Grade 3 and older MOs had clearly made a decision to stay long term, while Grade 1 and 2 MOs were still considering alternative career plans. Medical officers elaborated in detail on what made them feel supported in the workplace and this was clearly one of the stronger themes. The qualitative findings shed light on the factors influencing the overall rating of the facility (Figure 3) and being part of a cohesive team with good relationships was the strongest theme. Family and contextual factors also came out strongly as deal breakers in the decision to stay or leave, for example, living with one’s partner, providing for and parenting one’s children, and being close to family.

Overall, the district health services in the Western Cape appear to have established many of the foundational ingredients for retention and job satisfaction, such as good salaries and benefits, adequate equipment and supply chain and good infrastructure. Many studies have shown that once the salary is adequate, other factors are more important in retaining MOs.^15^ The staff numbers, mix of junior and senior clinicians, and turnover of team members were critical in terms of a manageable workload, team cohesion, supportive supervision, as well as professional development. Overall, staff numbers and mix appeared sufficient to ensure that these factors supported retention.

In terms of personal career intentions, there appeared to be a relationship between personal attributes, the scope of practice and retention. Those who were more attracted by a constant variety of work experiences across multiple disciplines were a better fit than those who preferred one specific area or were averse to some areas of the work, particularly in the district hospitals. Retention also appeared to be related to future expectations of career progression. Those who expected a future of relentless workload, constant overtime and performing the same tasks, albeit with increasing competency, seemed more likely to eventually leave. One of the implications was that the nature of the work should evolve as MOs gained seniority with, for example, the possibility of informal subspecialisation in a particular area or performing overtime from home to cover junior colleagues.^15^

The intensity and volume of the workload are identified as important factors in job satisfaction^15^ and the sense was that the workload was just manageable. Previous studies have identified burnout as prevalent amongst both rural and urban MOs in the Western Cape.^23,24^ Workload, therefore, is one of the key issues to proactively manage. Most MOs felt that their work was meaningful, recognised and appreciated, which is likely to provide intrinsic motivation.^14^ There were some concerns with a lack of recognition by peers working in other parts of the health system and from the public, which has been linked to reduced job satisfaction.^15^

Most MOs expressed job satisfaction related to working in a supportive and cohesive team and this was maybe the strongest theme that encouraged retention. This also implies that a lack of support or dysfunctional team would be a key factor in leaving. Supportive supervision from family physicians and managers was highlighted and there appeared to be significant improvement in this over the last few years, particularly at district hospitals. Previous studies have suggested an unsupportive organisational culture and reliance on command and control styles of management.^25^ This study suggests a move towards more collaborative leadership styles had occurred, which encouraged retention of MOs. The reasons for this shift are not clear, but could relate to leadership and management training in the department of health, as well as the employment of family physicians as managers in many facilities.

In addition, MOs reported the development of a strong learning environment with workplace-based clinical training and teaching and opportunities to attend external courses. Learning and quality improvement were part of the culture, and this was role modelled by family physicians and emulated by MOs. Opportunities for professional and personal development are recognised as key factors supporting retention.^15^ Outreach from specialists at the referral hospitals was appreciated and contributed to a stimulating learning environment, as has been shown in other studies.^26^

Interestingly, there was little intention to specialise in family medicine, although widespread appreciation for the family physicians in their teams. Specialisation in family medicine could provide an opportunity for career development while remaining in the district health services. Human resource analysis of family medicine has found the need for creating more public sector posts and for resolving the discrimination against such specialists within the private sector.^27^ Medical officers confirmed the contribution of family physicians to strengthening district health services and creating a positive work environment as clinicians and consultants, clinical trainers and capacity builders, as well as leaders of clinical governance. The attitudes towards private practice suggested that the perceived disadvantages outweighed the advantages: a finding in contradiction to the maldistribution of family doctors in private practice.^27^ It is possible that improvements in the working environment in the public sector of the Western Cape have tipped the balance more in favour of the public sector.

The personal, family and community issues were powerful in influencing retention and yet also the factors most outside the control of health services. Studies on rural and remote settings have emphasised the importance of employment opportunities for the spouse, educational opportunities for the children and being close to family.^28^ In the Western Cape context, it was important to be with your partner, to have family nearby, to enjoy social and recreational activities in the community, and to not have a long commute. There were no concerns expressed with basic living conditions, and safety was not seen as a substantial issue influencing retention.

### Limitations

All the MOs worked in facilities with family physicians and we do not know what the findings would be in facilities without family physicians. In the Western Cape, most of the district hospitals and community health centres have family physicians and MOs were from all districts in both urban and rural locations. The findings may, therefore, be transferable to the majority of district health services in this province. The Western Cape, however, performs better in many aspects of service delivery when compared with other provinces in South Africa, and the findings are likely to be different in other provinces.^29^

The interviews were conducted by family physicians within the SUFPREN network, and power dynamics could have influenced some of the responses. However, the MOs did not know the family physician who interviewed them and did not appear to have a social desirability bias in their responses, as shown by their honesty with regard to careers in family medicine. If a social desirability bias was present then respondents might have given more positive and favourable feedback about their work environment and the contribution of the family physician. It is also possible that family physicians put forward MOs for selection who they thought might be more positive about their facility or amenable to be interviewed, although they had to also select people they thought were likely to leave. Respondent validation with the MOs themselves was not attempted and could have added value to the analysis. A peer review by the family physicians who conducted the interviews allowed them to give feedback on whether the analysis by R.M. adequately interpreted the data they had collected.

Each interview was conducted by a different family physician, and there was a possibility for loss of fidelity or quality in the interviews. The first author, however, who listened to all the recordings, observed good fidelity to the interview guide and adequate interviewing skills. The first author performed the data analysis alone, but the interpretation was peer reviewed by the family physicians in a workshop.

Although community service MOs were excluded, because they were not in permanent positions, it might have been useful to explore their perspectives on staying employed within the district health services, even if not at the same facility. This could be the focus of another study.

The themes did not clearly differ between those staying and going. The impression was that MOs were actually on a spectrum from staying to going and were influenced by the same issues, particularly in the more junior grades. The motivation to finally stay or go may relate more to the strength and combination of these factors in a specific individual and their context.

### Recommendations

There is no single magic bullet for retaining MOs and a ‘bundle of interventions’ is probably required.^11^ Recommendations for retaining MOs are as follows:

- Ensure that the foundational elements are in place – adequate salary and benefits, adequate medication and supply chain, good infrastructure, sufficient staff numbers, and a combination of junior and senior clinicians
- Deal promptly with anything that undermines team dynamics and relationships, as working in a close, supportive and cohesive team is maybe the most important issue in the workplace.
- Having a family physician helps to retain MOs through supportive supervision, opportunities for professional development, a strong learning environment and support for improving the quality of care. Supportive supervision for more junior MOs is essential in building confidence and avoiding negative emotional impact from adverse events and errors.
- A shift from authoritarian and controlling management styles is important towards more appreciative, collaborative and supportive styles.
- Creating stronger career pathways within the district health services where people feel able to evolve and grow is important. This may mean differentiating the nature of work between grade 1 and grade 3 MOs and making specialisation in family medicine more attractive. The Postgraduate Diploma in Family Medicine can also form part of this career pathway and progression and the district health services (DHS) are already starting to support MOs in this Diploma.
- Create flexible regulations for working hours and overtime that support MOs when they become parents and need to balance work and family responsibilities.^30^
- Realise that contextual issues may be deciding factors, but out of your control, for example, moving to be with a life partner, close to family, close to work or in an attractive community. On the other hand, it has been observed that a multisectoral approach in rural areas could tackle some of the key contextual issues.^11^
- Proactively monitor and manage the workload to ensure it remains manageable. If the workload is constantly too intense or too high then this may lead to burnout and loss of staff.

## Conclusion

Retention of MOs in district health services was linked to personal career intentions, their experience of clinical work, their experience of the organisation, as well as personal, family and community issues. Junior MOs saw the district health services as a good place to learn skills and from which to decide on future career plans. Those who enjoyed the wide variety of clinical work were more likely to feel comfortable remaining. Most were not considering specialising in family medicine, which would retain them in the district health services, while appreciating the contribution of family physicians to their work experience. Having the opportunity for career progression that went beyond an increase in salary might encourage people to remain in the organisation. Few were attracted by the private sector. Being part of a close and cohesive team was the strongest factor in retaining MOs. Having supportive clinical supervision and working in a positive learning environment were also important. An appreciative, collaborative and supportive management style was also influential in retaining MOs. Management that was flexible to meet the evolving needs of MOs, particularly for childcare and parenting, was needed. A foundation of functioning supply chain, good salaries and benefits, adequate staff numbers and mix, and good infrastructure were present. Being with your partner and close to family and living close to work were important factors. Being in a community with educational, social and recreational resources was influential. Leaders and managers of the healthcare services should intervene across multiple factors to create the conditions needed to retain MOs.
